# Supplementary material for: A burning issue: Reviewing the socio-demographic and environmental justice aspects of the wildfire literature
Source: PLoS One. 2022 Jul 28;17(7):e0271019. doi: 10.1371/journal.pone.0271019 (PMC9333234; doi:10.1371/journal.pone.0271019)

**Supporting Information 1 Figure.** A dendrogram developed using Bibliometrix and author provided keywords showing the topic distribution of publications (n = 299) included in the literature review. Topics associated with wildfire smoke and air pollution in the blue branch, and subsequent effects, are a distinct branch that is separate from the wildfire and socio-demographic topics (shown in the red branches) addressed in this study.


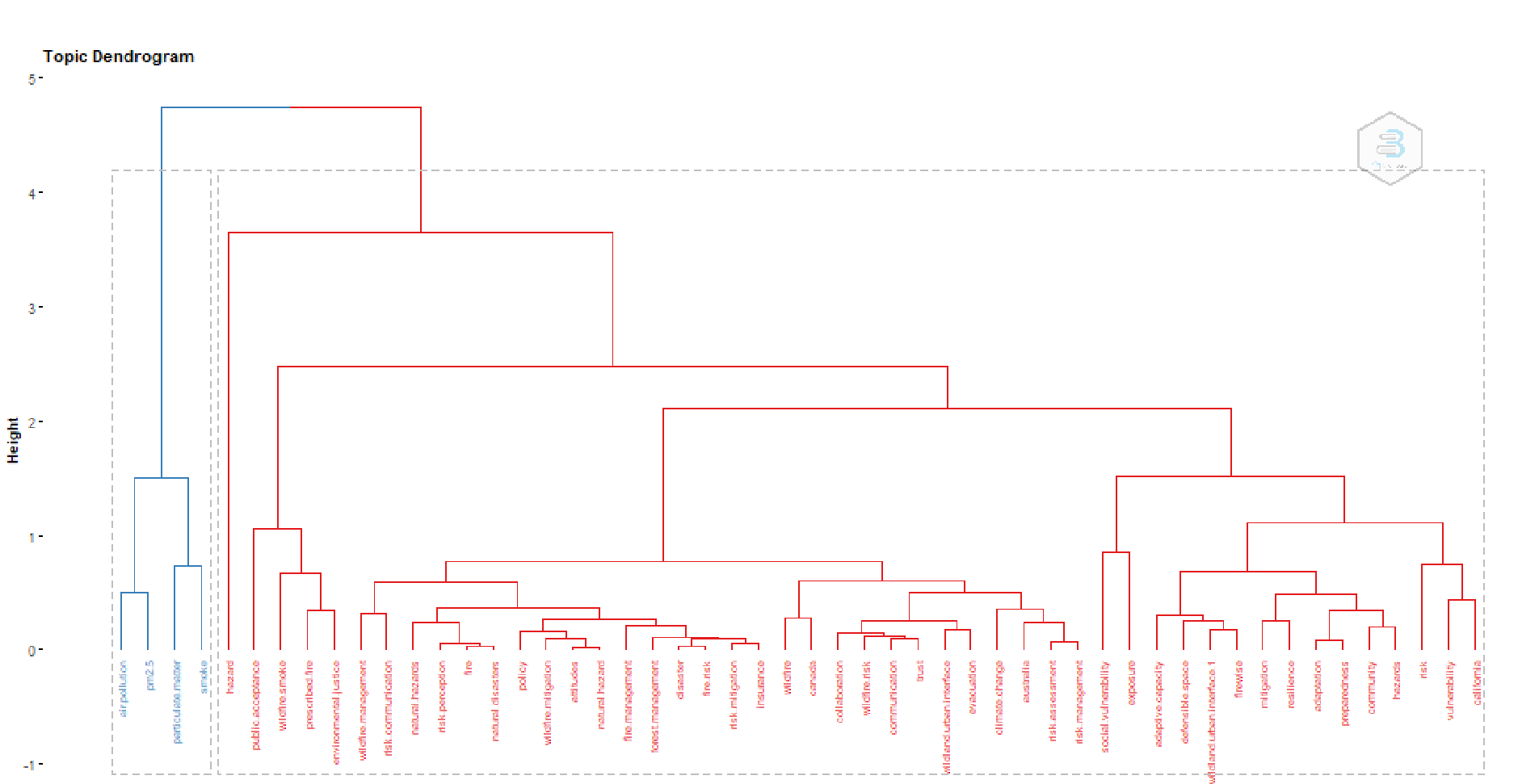

Supplement: S1 Fig — Topics associated with wildfire smoke and air pollution in the blue branch, and subsequent effects, are a distinct branch that is separate from the wildfire and socio-demographic topics (shown in the red branches) addressed in this study. (DOCX) [file pone.0271019.s001.docx]
