# Supplementary material for: A burning issue: Reviewing the socio-demographic and environmental justice aspects of the wildfire literature
Source: PLoS One. 2022 Jul 28;17(7):e0271019. doi: 10.1371/journal.pone.0271019 (PMC9333234; doi:10.1371/journal.pone.0271019)
Supplement: S1 Table — Note: Df, Degrees of Freedom; Resid., Residual; Pr, Probability. (DOCX) [file pone.0271019.s002.docx]

**Supporting Information 4 Table.** Analysis of deviance table for the probit model for variables significantly related to the classification of a publication as relevant to our definition of Environmental Justice. Note: Df, Degrees of Freedom; Resid., Residual; Pr, Probability

| Variable | Df | Deviance | Resid. Df | Resid. Dev | Pr(>Chi) | Significance |
| --- | --- | --- | --- | --- | --- | --- |
| Year | 1 | 14.8921 | 297 | 284.9 | 0.0001138 | *** |
| Community | 6 | 23.8558 | 291 | 261.05 | 0.0005551 | *** |
| Age | 1 | 3.1871 | 290 | 257.86 | 0.0742234 | . |
| Sex | 1 | 0.2587 | 289 | 257.6 | 0.61103 |  |
| House | 1 | 11.1257 | 288 | 246.47 | 0.0008514 | *** |
| Language | 1 | 3.9955 | 287 | 242.48 | 0.045622 | * |
| Race | 1 | 16.3913 | 286 | 226.09 | 5.15E-05 | *** |
| Poverty | 1 | 26.7308 | 285 | 199.36 | 2.34E-07 | *** |
| Education | 1 | 0.6289 | 284 | 198.73 | 0.4277706 |  |
| Pre-fire | 1 | 1.2351 | 283 | 197.49 | 0.2664237 |  |
| During fire | 1 | 0.156 | 282 | 197.34 | 0.6929101 |  |
| Post-fire | 1 | 5.0946 | 281 | 192.24 | 0.0240003 | * |
| Hazard-fire | 1 | 0.3882 | 280 | 191.85 | 0.5332273 |  |
| Hazard-smoke | 1 | 2.3974 | 279 | 189.46 | 0.1215396 |  |
| Hazard-other | 1 | 0.0189 | 278 | 189.44 | 0.8906957 |  |
| USA | 1 | 5.7659 | 277 | 183.67 | 0.0163403 | * |
| Canada | 1 | 0.5712 | 276 | 183.1 | 0.4497815 |  |
| Australia | 1 | 2.596 | 275 | 180.5 | 0.1071318 |  |

Significance: *** 0.001; ** 0.01; * 0.05
