# Supplementary material for: A burning issue: Reviewing the socio-demographic and environmental justice aspects of the wildfire literature
Source: PLoS One. 2022 Jul 28;17(7):e0271019. doi: 10.1371/journal.pone.0271019 (PMC9333234; doi:10.1371/journal.pone.0271019)
Supplement: S1 File — (DOCX) [file pone.0271019.s003.docx]

**Supporting Information 1 File. Background of environmental justice based on policies from the United States**

In the United States for example, its Environmental Protection Agency defines EJ as “the fair treatment and meaningful involvement of all people regardless of race, color, national origin, or income, with respect to the development, implementation, and enforcement of environmental laws, regulations, and policies. This goal will be achieved when everyone enjoys the same degree of protection from environmental and health hazards, and equal access to the decision-making process to have a healthy environment in which to live, learn, and work” [1]. Adam and Charnley [2], who studied the EJ of hazardous fuel treatments in US National Forests, categorized the concept into three themes: (1) social vulnerability and resilience in relation to wildfire risk management; (2) EJ and federal agency natural resource management; and (3) participation of EJ populations in collaborative resource management by US federal agencies.

Policy-wise, since 1994 each US government agency has been required to make “environmental justice part of its mission by identifying and addressing, as appropriate, disproportionately high and adverse human health or environmental effects of its programs, policies, and activities on minority populations and low-income populations” (§1-101, 59 Fed. Reg. 7629, 1994). However, in 2004 the emphasis was changed by removing the focus on minority and low-income populations to promote EJ for everyone [3]. At the time of writing, the current administration has made EJ a priority and defines these communities as “geographic location[s] with significant representation of persons of color, low-income persons, indigenous persons, or members of Tribal nations, where such persons experience, or are at risk of experiencing, higher or more adverse human health or environmental outcomes” [4].

**References**

1. Environmental Protection Agency [EPA]. Environmental Justice. 2021 [cited 9 December 2021]. Available from: https://www.epa.gov/environmentaljustice

2. Adams MD, Charnley S. The environmental justice implications of managing hazardous fuels on federal forest lands. Ann Am Assoc Geog. 2020 Nov 1

3. Environmental Protection Agency Office of the Inspector General [Inspector General]. EPA needs to consistently implement the intent of the executive order on environmental justice. 2004 [cited 9 December 2021]. Available from: <https://www.epa.gov/sites/default/files/2015-12/documents/20040301-2004-p-00007.pdf>.

4. White House Environmental Justice Advisory Council. Final Recommendations: Justice40, Climate and Economic Justice Screening Tool & Executive Order 12898
