# Supplementary material for: A burning issue: Reviewing the socio-demographic and environmental justice aspects of the wildfire literature
Source: PLoS One. 2022 Jul 28;17(7):e0271019. doi: 10.1371/journal.pone.0271019 (PMC9333234; doi:10.1371/journal.pone.0271019)
Supplement: S2 File — (DOCX) [file pone.0271019.s004.docx]

**Supporting Information 2 File: Table and detailed methodological information on analytical methods, models, variables, and software**

*S2.1 Literature review variables and entry codes*

S2 Table. Literature review categories of attributes, their possible coding entries, and relevant guidelines and examples. These categories were used to code the 299 final documents selected for inclusion in this analysis. N/A was used for publications that used models or other methods not specific to a state/province/community type.

| **Review variable** | **Possible entries** |
| --- | --- |
| Country | Not limited |
| State/Province (if USA, Canada, or Australia) | Any state(s) or province(s), all, N/A |
| Community type(s) | Rural, WUI, urban, urban and WUI, rural and WUI, all, N/A |
| Time period | Pre wildfire/prescribed fire, during wildfire/prescribed fire, post-wildfire, modeled wildfire |
| Hazard(s) | Wildfire, smoke, fire effects (e.g., mudslides), other (e.g., prescribed fire) |
| Environmental justice (EJ) | Yes or no overall. If yes, then yes or no for 4 components: Impacts/harm, information/governance, amenities, justice/remedies. |
| Socio-demographics | Age, gender, education, employment, ethnicity/race, housing, income, language, poverty, other, none |

*S2.2. Bibliometric and Statistical Analyses and Methodological Details*

Once all publications were coded and QA/QC checks were completed, systematic and quantitative analyses were also carried out using R Version 4.0.2 [1] and the package Bibliometrix [2] and its online interface Biblioshiny [2]. These analyses included summarizing key fields such as source, year of publication, and country of author affiliations; and chi-squared test for the relationship between EJ designation and the specific socio-demographic variables. Other bibliometric analyses were carried out using VOS viewer version 1.6.17 [3], a free software program that detects the intellectual structure of a research topic and maps the chosen field (e.g., author, keyword, country of authors) onto a network made up of multiple clusters. These networks can be based on co-authorship, co-occurrence, citation, or bibliographic coupling links.

To further explore what factors were important for determining whether a paper was classified as addressing EJ issues, we used R version 4.0.2 [1] to construct the following probit regression model that predicted EJ designation:

${prob(EJ}_{i}={1|Y_{i},W_{i},S_{i},P_{i},H_{i,}C_{i})=\beta_{1}\beta}_{1}Y_{i}+\mu W_{i}+\gamma S_{i}+\alpha P_{i}+\delta H_{i}+\rho C_{i}+\varepsilon_{i}$

where ${EJ}_{i}$ is a binary variable which equals 1 if the paper was classified as addressing EJ according to our definition, and 0 otherwise, $Y_{i}$ is a continuous time trend, $W_{i}$ is a categorical variable for the type of community the study considers, $S_{i}$ is a set of binary socio-demographic variables for whether a study includes age, sex, house, language, race, and education which equal 1 if the study included them, and 0 otherwise. $P_{i}$ is a set of binary variables indicating which period the study considers, and includes pre-fire, during a fire, and post-fire. $H_{i}$ is a set of variables that contain the kinds of effects the paper considered such as: wildfire, smoke, fire effects, etc. Finally, $C_{i}$ is a set of three binary variables for whether the paper considered the US, Canada, or Australia and the error term $\varepsilon_{i}$ is assumed to be normally distributed. Additionally, chi square tests in R Version 4.0.2 [1] were used to test EJ designation and its relationship to commonly used socio-demographic variables.

Finally, we also used author keywords to plot the collection themes into four quadrants depending on centrality and density rank values. According to Cobo et al. [4], the centrality measures the degree of interaction of a network with other networks as “a measure of the importance of a theme in the development of the entire research field analyzed”. The density measures the internal strength of the network and identifies the degree of development of a theme. The density is measured by the number of connections (or edges) of a given node to another, over the number of possible connections (i.e., edges). Thus, the cluster size reflects the frequency of the keywords in the document set.

**References**

1. R Core Team. 2020. R: A Language and Environment for Statistical Computing. Vienna, Austria: R Foundation for Statistical Computing. <https://www.R-project.org/>.

2. Aria M, Cuccurullo C. Bibliometrix: An R-tool for comprehensive science mapping analysis. J Informetr. 2017 Nov 1;11(4):959-75. doi: 0.1016/j.joi.2017.08.007.]

3. Van Eck NJ, Waltman L. Citation-based clustering of publications using CitNetExplorer and VOSviewer. Scientometrics. 2017 May 1;111(2):1053-70.

4. Cobo MJ, López-Herrera AG, Herrera-Viedma E, Herrera F. An approach for detecting, quantifying, and visualizing the evolution of a research field: A practical application to the fuzzy sets theory field. J Informetr. 2011 Jan 1;5(1):146-66. doi: 10.1016/j.joi.2010.10.002.
