# Supplementary material for: A burning issue: Reviewing the socio-demographic and environmental justice aspects of the wildfire literature
Source: PLoS One. 2022 Jul 28;17(7):e0271019. doi: 10.1371/journal.pone.0271019 (PMC9333234; doi:10.1371/journal.pone.0271019)

**Supporting Information 3 File: Additional results including text, figures, and tables.**

*S3.1 Socio-demographic attributes*

Supporting Information 3 Table 3A. Socio-demographic attributes of the wildfire literature. Please refer to Table 2 for complete descriptions and guidelines.

| **Category** | **Attribute analyzed** | **% of documents** |
| --- | --- | --- |
| Community type (n=299) | Rural | 10% |
|  | Rural and WUI | 7% |
|  | WUI | 44% |
|  | Urban and WUI | 0.3% |
|  | Urban | 2% |
|  | All | 29% |
|  | N/A | 7% |
| Time period of study (n=299) | Pre-wildfire/prescribed fire | 68% |
|  | During wildfire/prescribed fire | 20% |
|  | After wildfire | 29% |
| Hazard (n=299) | Wildfire | 82% |
|  | Smoke | 11% |
|  | Post-wildfire events^a^ | 3% |
|  | Fuel treatments^b^ | 18% |
| Socio-demographics (n=180) | Age | 56% |
|  | Income | 54% |
|  | Gender | 42% |
|  | Education | 40% |
|  | Housing | 34% |
|  | Employment | 31% |
|  | Ethnicity/Race | 31% |
|  | Poverty | 19% |
|  | Language | 11% |
|  | Other^c^ | 51% |

^a^ Post-wildfire events include flooding and debris flows.

^b^ Fuel treatments include prescribed fire and mechanical thinning.

^c^ Other includes part-time or full-time residency, length of residency, physical disability, and home ownership.

*S3.2 Journals*

The documents were published in 110 different journals in the fields of natural disasters, the environment, forestry, sociology, health, and economics. However, a sizeable percentage (35%) of the publications considered came from just six journals: International Journal of Wildland Fire, Society and Natural Resources, Environmental Management, Journal of Forestry, International Journal of Disaster Risk Reduction, and Natural Hazards (S3 Table 3B). Furthermore, only 25 journals (24%) had published more than two articles, whereas 81 journals (76%) had published only one article.

Supporting Information 3 Table 3B: Top ten journals with the greatest number of documents on the social-demographic aspects of wildfire.

| **Journal** | **Number of documents** |
| --- | --- |
| International Journal of Wildland Fire | 26 |
| Society and Natural Resources | 21 |
| Journal of Forestry | 19 |
| Environmental Management | 18 |
| International Journal of Disaster Risk Reduction | 14 |
| Natural Hazards | 8 |
| Journal of Environmental Management | 7 |
| Landscape and Urban Planning | 7 |
| Science of the Total Environment | 7 |
| Environmental Hazards | 6 |

*S3.2 Geographical scope, contexts, and collaborations*

Country-level scientific production, as measured by country of author affiliations, was represented 28 countries (S3Table 3C). However, it was dominated by the United States, with 745 appearances by US authors, and Australia was a distant second with 147 appearances of Australian authors. Canada had 105, followed by Spain (26), Portugal (19) and Chile (12) were the only other countries with more than 10 authors. Supporting Information Table 3D shows the geographic area or focus of the publications. Refer to Supporting Information Tables 3E and 3F for a detailed breakdown of the state/provincial breakdown of the research in the United States, Canada and Australia, respectively.

Supporting Information 3 Table 3C. Country level scientific production, as measured by author affiliations

| **Country** | **Author appearances** |
| --- | --- |
| United States | 745 |
| Australia | 147 |
| Canada | 88 |
| Spain | 26 |
| Portugal | 19 |
| Chile | 12 |
| Italy | 10 |
| United Kingdom | 9 |
| France | 6 |
| Germany | 6 |
| Switzerland | 5 |
| Thailand | 5 |
| Mexico | 4 |
| Algeria | 3 |
| Argentina | 3 |
| Greece | 3 |
| Israel | 3 |
| Sri Lanka | 3 |
| Finland | 2 |
| Ivory Coast | 2 |
| Norway | 2 |
| Belize | 1 |
| Brazil | 1 |
| China | 1 |
| New Zealand | 1 |
| Poland | 1 |
| Singapore | 1 |
| South Africa | 1 |

Supporting Information 3 Table 3D. Geographic area of focus of publications.

| **Country** | **Number of publications** |
| --- | --- |
| Algeria | 1 |
| Argentina | 1 |
| Australia | 47 |
| Brazil | 1 |
| Canada | 26 |
| Chile | 4 |
| Corsica | 1 |
| France | 1 |
| Greece | 1 |
| Italy | 3 |
| Ivory Coast | 1 |
| Mexico | 1 |
| New Zealand | 1 |
| Norway | 1 |
| Portugal | 4 |
| Spain | 6 |
| Thailand | 1 |
| United States | 195 |
| Global (33 countries) | 1 |

Supporting Information 3 Table 3E. Location of publications specifically researching or discussing the United States. Note that percentages do not equal 100 as some publications covered multiple states.

| **State** | **Number of Publications (Percentage)** | **State** | **Number of Publications (Percentage)** |
| --- | --- | --- | --- |
| California | 51 (26%) | Wyoming | 4 (2%) |
| Oregon | 48 (25%) | Kentucky | 3 (2%) |
| Colorado | 44 (23%) | Mississippi | 3 (2%) |
| Montana | 26 (13%) | Pennsylvania | 3 (2%) |
| Washington | 24 (12%) | Arkansas | 2 (1%) |
| Florida | 21 (11%) | Hawaii | 2 (1%) |
| Idaho | 20 (10%) | Maine | 2 (1%) |
| Arizona | 17 (9%) | Missouri | 2 (1%) |
| New Mexico | 15 (8%) | Iowa | 1 (0.5%) |
| Utah | 13 (7%) | Kansas | 1 (0.5%) |
| Alaska | 12 (6%) | New Hampshire | 1 (0.5%) |
| Minnesota | 12 (6%) | New Jersey | 1 (0.5%) |
| Nevada | 12 (6%) | New York | 1 (0.5%) |
| Texas | 11 (6%) | North Dakota | 1 (0.5%) |
| Georgia | 10 (5%) | Ohio | 1 (0.5%) |
| Michigan | 8 (4%) | South Dakota | 1 (0.5%) |
| Alabama | 7 (4%) | West Virginia | 1 (0.5%) |
| North Carolina | 7 (4%) | Connecticut | 0 (0%) |
| Oklahoma | 7 (4%) | Delaware | 0 (0%) |
| South Carolina | 7(4%) | Illinois | 0 (0%) |
| Wisconsin | 6 (3%) | Indiana | 0 (0%) |
| Louisiana | 5 (3%) | Maryland | 0 (0%) |
| Virginia | 5 (3%) | Nebraska | 0 (0%) |
| Massachusetts | 4 (2%) | Rhode Island | 0 (0%) |
| Tennessee | 4 (2%) | Vermont | 0 (0%) |
| All or N/A | 18 (9%) | **Total** | 195 |

Supporting Information 3 Table 3F. Location of publications specifically researching or discussing Canada or Australia. Note that percentages might not equal 100 as some publications covered multiple states/territories.

| **Canada** | |  | **Australia** | |
| --- | --- | --- | --- | --- |
| **Province** | **Number of Publications (Percentage)** |  | **State** | **Number of Publications (Percentage)** |
| Alberta | 12 (46%) |  | Victoria | 17 (36%) |
| Ontario | 5 (19%) |  | New South Wales | 13 (28%) |
| British Columbia | 3 (12%) |  | South Australia | 5 (11%) |
| Northwest Territories | 1 (4%) |  | Queensland | 4 (9%) |
| Saskatchewan | 1 (4%) |  | Australian Capital Territory | 3 (6%) |
| Quebec | 1 (4%) |  | Western Australia | 3 (6%) |
| Manitoba | 0 (0%) |  | Tasmania | 1 (2%) |
| New Brunswick | 0 (0%) |  | South-East Australia | 1 (2%) |
| Newfoundland and Labrador | 0 (0%) |  | Northern Territory | 0 (0%) |
| Nova Scotia | 0 (0%) |  | All or N/A | 4 (9%) |
| Nunavut | 0 (0%) |  |  |  |
| Prince Edward Island | 0 (0%) |  |  |  |
| Yukon | 0 (0%) |  |  |  |
| All or N/A | 3 (4%) |  |  |  |
| **Total** | 26 |  | **Total** | 51 |

Supporting Information 3 Fig 3, maps the geographical focus of publications by state/province for the United States, Canada, and Australia and S3 Fig 4 provides an overview of the scientific collaboration between countries. Of the 28 countries that had at least one author, 22 had multi-country collaborations and could be mapped onto a network. The resulting network has seven clusters, with the United States, Australia, and Canada having the largest nodes. All multi-country nodes spanned more than one continent.

Supporting Information 3 Figure 3. Geographical focus of publications by state/province for the A) United States, B) Canada, and C) Australia. Note: The map of Australia was accessed from the Australian Bureau of Statistics [https://www.abs.gov.au/statistics/standards/australian-statistical-geography-standard-asgs-edition-3/jul2021-jun2026/access-and-downloads/digital-boundary-files/STE_2021_AUST_SHP_GDA2020.zip](https://gcc02.safelinks.protection.outlook.com/?url=https%3A%2F%2Fwww.abs.gov.au%2Fstatistics%2Fstandards%2Faustralian-statistical-geography-standard-asgs-edition-3%2Fjul2021-jun2026%2Faccess-and-downloads%2Fdigital-boundary-files%2FSTE_2021_AUST_SHP_GDA2020.zip&data=04%7C01%7C%7C3a66303d7bde413abf2908da136be07d%7Ced5b36e701ee4ebc867ee03cfa0d4697%7C0%7C0%7C637843651292824017%7CUnknown%7CTWFpbGZsb3d8eyJWIjoiMC4wLjAwMDAiLCJQIjoiV2luMzIiLCJBTiI6Ik1haWwiLCJXVCI6Mn0%3D%7C3000&sdata=ZjDGb5xCZsFTMvuVp%2BGdCyraZZkme9MtjnzdbM5Cw2c%3D&reserved=0). The map of Canada was accessed from the Government of Canada’s Open Data website [https://open.canada.ca/data/en/dataset/a883eb14-0c0e-45c4-b8c4-b54c4a819edb](https://gcc02.safelinks.protection.outlook.com/?url=https%3A%2F%2Fopen.canada.ca%2Fdata%2Fen%2Fdataset%2Fa883eb14-0c0e-45c4-b8c4-b54c4a819edb&data=04%7C01%7C%7C3a66303d7bde413abf2908da136be07d%7Ced5b36e701ee4ebc867ee03cfa0d4697%7C0%7C0%7C637843651292824017%7CUnknown%7CTWFpbGZsb3d8eyJWIjoiMC4wLjAwMDAiLCJQIjoiV2luMzIiLCJBTiI6Ik1haWwiLCJXVCI6Mn0%3D%7C3000&sdata=0vQjFaIB%2BXumdayqyUAyxhG%2BrLqFGFV2NBp9XoT36jg%3D&reserved=0) and is covered under the open government license – Canada. The state map of the United States was accessed through the United States Census Bureau’s cartographic boundaries page[https://www.census.gov/geographies/mapping-files/time-series/geo/carto-boundary-file.html](https://gcc02.safelinks.protection.outlook.com/?url=https%3A%2F%2Fwww.census.gov%2Fgeographies%2Fmapping-files%2Ftime-series%2Fgeo%2Fcarto-boundary-file.html&data=04%7C01%7C%7C3a66303d7bde413abf2908da136be07d%7Ced5b36e701ee4ebc867ee03cfa0d4697%7C0%7C0%7C637843651292824017%7CUnknown%7CTWFpbGZsb3d8eyJWIjoiMC4wLjAwMDAiLCJQIjoiV2luMzIiLCJBTiI6Ik1haWwiLCJXVCI6Mn0%3D%7C3000&sdata=P253a80MYVd5bn9fJvo%2BcJYTV9saY%2BgJNJqnm3hfk%2Fo%3D&reserved=0)

**
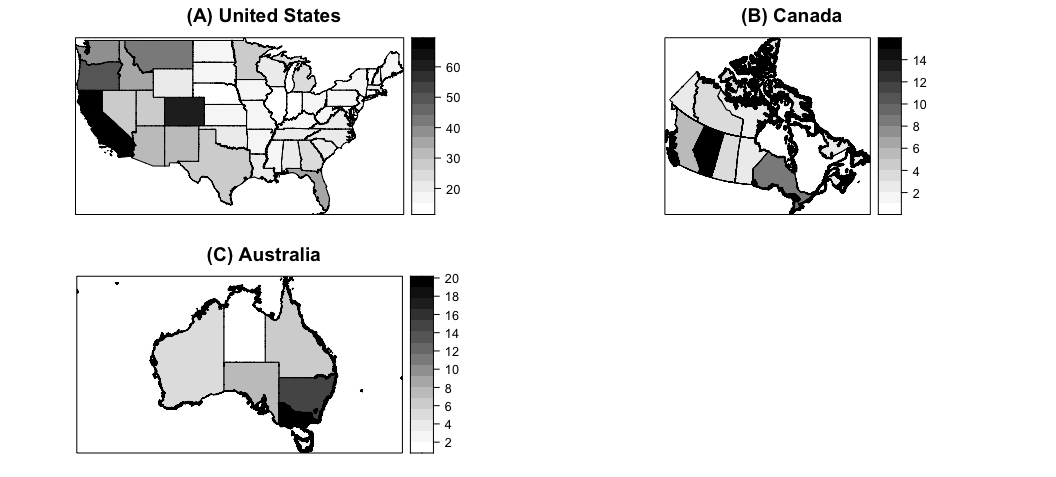
**

Supporting information 3 Figure 4. Collaboration network of different countries based on author affiliations. There are 34 links, with a total strength of 60. Numbers refer to the different clusters.


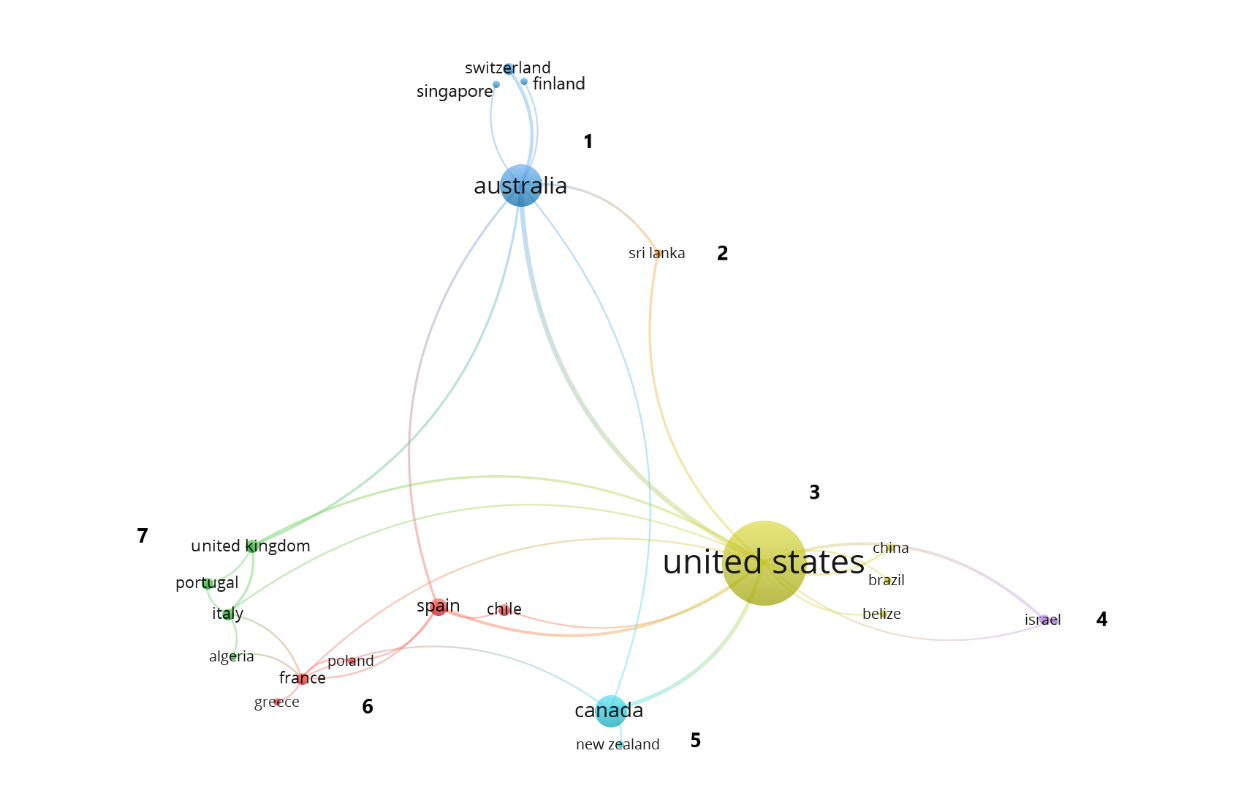

Supplement: S3 File — (DOCX) [file pone.0271019.s005.docx]
