# Supplementary material for: A burning issue: Reviewing the socio-demographic and environmental justice aspects of the wildfire literature
Source: PLoS One. 2022 Jul 28;17(7):e0271019. doi: 10.1371/journal.pone.0271019 (PMC9333234; doi:10.1371/journal.pone.0271019)
Supplement: Attachment — Submitted filename: PONE_Response to reviewers_R2.docx [file pone.0271019.s007.docx]

RESPONSE TO REVIEWERS

Additional Editor Comments:

Please make the following changes to your paper:
1) your findings, and the reviewer comments, seem to point to several important findings that could be more clearly communicated in the abstract, introduction, and discussion. These relate to the four connected themes you addressed: wildfires, the wildland-urban interface, environmental justice, and health.

- Over the past few decades, wildfire behavior has changed: formerly wildfires tended to be confined to natural vegetation (forests, scrublands, grasslands), but increasingly they are impacting residential rural, peri-urban, and urban areas.
- This means that wildfire impacts on people are no longer confined to the wildland-urban interface, defined as a zone far removed from urban areas, and instead wildfires are affecting rural, suburban, and urban areas.
- In turn, this means that multiple different groups of vulnerable people are being affected by wildfire, including (a) poor, white, underinsured people living in depressed rural towns such as former mining, forestry, or other areas, (b) marginalized groups living in peri-urban or urban settings where dwellings are vulnerable, such as migrant worker trailer parks, and (c) marginalized groups living in urban areas who may be disproportionately affected by smoke from wildfires because of a lack of capacity to adapt to these conditions.
- Thus, wildfire now poses a wide range of threats to livelihood, life, and health across all rural, suburban, and urban areas. The literature that you review does not appear to have yet adapted to this new reality, and it would strengthen your paper if you could emphasize these gaps and the need for more work in these topics.

*Thank you for summarizing our study. Indeed,* *we have taken these 4 themes to heart and have reorganized our Discussion and much of our Conclusion into 3 sections. The first section is where we discuss our findings relative to our 3 stated objectives. This has served to focus our discussion and to not tangentially delve into disparate topics. We then have a distinct limitations paragraph. And we then have reorganized and presented the end of our discussion text around a distinct Future Research section which is organized according to the 4 themes your highlighted. Finally, we close with a short succinct 2 paragraph Conclusion. As you will see we have also revised our Introduction following your indications and that of other reviews. We have also emphasized the use of our proposed wildfire relevant EJ definition in formulating environmental policies.*

2) your manuscript is currently almost 10,500 words long. To increase the readability and impact of your paper, please shorten it to 6000-7000 words. Some portions of text could be moved into a supplement.

*We have eliminated repetitive text, nonrelevant information and moved entire sections to Supporting Information. This has reduced the word count in the main text (i.e., Introduction to Conclusion) to 7, 338 words. We have also eliminated 12 references that were comprised of approximately 340 words (these references were unnecessary or repeating other references). As requested, we have also moved Background on US policy on Environmental justice, literature review categories, and bibliometric and statistical analyses methods; plus journal and geographical scope, contexts, and collaborations results to the Supporting Information sections. We have also consolidated and reduced the number of supporting information files.*

3) I note that the text contains a Table 1, Table 2, and Table 6. Please adjust/correct, and check the figure numbers also.

We apologize for this mistake. This is in large part due to our use of Supporting Information materials. However, we have changed Table 6 to Table 2:

“both of which increased the likelihood that a study was EJ (Table 2)” lines 368-369

“**Table 2**. Results of the probit model” line 375

“have a lower likelihood of being labeled as EJ (Table 2).” Line 487

We have also double checked the other figure, table, and text numbers in the text and Supporting Information section for consistent and accurate numbering.

If you are able to make these changes, it may not be necessary to send your paper out for a third round of reviews.

**Comments to the Author**

6. Review Comments to the Author

Please use the space provided to explain your answers to the questions above. You may also include additional comments for the author, including concerns about dual publication, research ethics, or publication ethics. (Please upload your review as an attachment if it exceeds 20,000 characters)

Reviewer #2: I am confused as to how the dendrogram in Supplemental Figure 1 negates the need for any mention of health literature. While it clearly shows the distinction between the air pollution topics and socio-demographic topics, many of the socio-demographic categories are related to health and should be treated as such. To define health as only having to do with the branch of air quality and wildfires is not accurate. The other reasonings presented to not include health were more compelling (saturation of the literature), however it still seems to be an oversight to omit health searches when they could be directly related to some of the other socio-demographics topics discussed. This should be mentioned in the discussion.

*You are correct in that health issues do influence social vulnerability. There are two subsets of the wildfire smoke literature. The first relates to exposure to the smoke, either wildfire or prescribed fire. The second would be the health effects of the smoke which is a different field targeted towards a different audience and published in different journals (as we previously mentioned). However, we agree with your suggestion to clearly link health and other socio-demographic topics. We have added the following text, “*Similarly, certain socio-demographic groups are more vulnerable to wildfire smoke effects due to factors such as pre-existing health conditions, and a lack of insurance/medical care. In Australia, effects of wildfire smoke on health tended to be stronger for Aboriginal populations (Hanigan et al., 2008).” lines 409-412. We have added a new reference accordingly (D’Evelyn et al., 2022) to further substantiate the saturation of the literature issue you raise.

Reviewer #3: In my original comments, I asked for more of an explanation of how EJ factors make people more vulnerable. Based on your response, it seems the primary groups impacted are Native Americans and sometimes the elderly. Most EJ groups don't live in fire prone areas, so the application of an EJ framing seems a bit of a stretch. The authors need to state more explicitly how EJ does affect Indians and elderly people, given they are more at risk.

*Thank you for providing the opportunity to address this point. It is true that two US communities that are at risk of wildfire related effects are the elderly and Native American groups; however, we argue that there are other socio-demographic groups across the globe that are affected by wildfire than those two. And this will depend on geographies, context, countries, and regions within those countries. We contend that EJ communities are present in fire prone areas –not only the US but across the globe- and that the number of EJ communities at risk continue to grow. In order to clarify all of these points and to address this comment, we added and edited language on Page 23, which now reads:*

*“Overall, studies from our review that focused on EJ described different impacts to, and the social vulnerability of different communities. For example, Masri et al. (2021) found that except for Native American communities, wildfires impact census tracts with lower populations of minorities and higher populations of elderly. Elderly populations are often dependent on external assistance for evacuation (Masri et al., 2021) and are also highly susceptible to smoke-related health conditions (Liu et al., 2015). Furthermore, the elderly population is more likely to have fixed incomes, making them more likely to relocate to peri-urban zones due to housing price increases (Murphy and Allard, 2015), and have limited capacity to address the financial hardships of a wildfire. In addition to being disproportionately located in areas of high fire risk, Native American communities also have high social vulnerability and low adaptive capacity (Davies et al., 2018); including high levels of poverty, low levels of education, and commonly experience household crowding.” lines 488-499*

Which authors are referenced here: “Conversely, these authors note that lower income and minority populations have been largely reported to be concentrated in areas historically at lower risk, such as in city cores and highly urbanized suburbs.”

*Wigtil et al. (2016) and Davies et al. (2018) as stated in the preceding sentence; hance “..these authors..”.*

……Amenity migration by higher income populations to peri-urban areas also puts existing residents, that are often vulnerable populations, at risk in those communities through a larger number of homes to defend in case of wildfire, since newer residents often choose not to engage in fuel reduction projects
(Collins, 2008).

*Collins, 2008 is referred to here as cited at the end of the text you mention.*

“through a….” needs to be reworded. Not the correct term

*We have rephrased this and it now reads, “…rather than strictly through a land, resource, or fire management lens..”*

However, we respectfully disagree with you regarding the use of “choices” and “positive terms”. The definition of Environmental Justice as used in this paper is a framework to understand the inherent lack of choices, and limitations, encountered by historically disadvantaged communities.
Again, this framing is too absolutist. People always have choices. You suggest that this cannot be the case ever, when in fact there is always some degree of agency. Your definition of EJ should be tempered.

*Thank you for your comments. We understand that there is always some degree of agency when studying human populations. But is “choice” really viable when considering injustices and people with no power or influence, and historical legacies of disenfranchisement? One could argue that people always can move to another home or area, but the reality is that many people cannot. Perhaps they cannot afford to move because all other areas near their employment are too expensive. Perhaps they depend on nearby family who live next door for childcare. Native Americans did not choose to live on reservations and given their high poverty and strong place attachment, where would they move to? There are many reasons why one simply cannot choose to move to a place at less risk. The very people who have more choices are usually the ones that are not highly vulnerable. We also note that nowhere in our definition does it say that these discrepancies are due to a lack of choices. “Inequitably” references to the outcome, not the process.*

*However, to address your point, we conclude our study, by stating, “In conclusion, it is our hope that our proposed definition of EJ can be used by both governmental and non-governmental organizations to guide the inclusion of EJ in their fire, land, and environmental policies and management efforts. However, care is warranted in that EJ should not be used as a quantitative or binary metric. Rather, EJ can be better used as a lens or framework to understand the impacts of emerging environmental problems affecting diverse and often disadvantaged communities.” Lines 631-636*

Reviewer #4: The manuscript is much improved. However there are some opportunities for further improvement.

While the authors seem to have taken my questions and suggestions as personal attacks, my suggestions were aimed at improving the manuscript so that it could reach and speak to a broader audience, and thereby have an improved impact. I did not appreciate the tone of the authors responses to my comments. For example, my observation of use of forestry jargon could be interpreted to mean that the non-forestry audience reading PlosOne would not comprehend the content of this manuscript. PlosOne is not a forestry journal, in fact it is explicitly a multi-disciplinary journal, and therefore publishing in this journal is an opportunity to reach broader audiences. I suggest that the authors complete their revision/response, put it aside, and then return to it with an eye towards whether they have responded in a constructive and respectful manner. Another option is to ask an experienced colleague to review their response document prior to submitting.

*We apologize for appearing to take this as a personal attack. We did not I assure you. What we were doing is simply responding in-kind to the tone you were using. For example, we know that PLOS One is not a forestry journal; in fact, most of the interdisciplinary co-authors have previously published before in this journal. And having used the term critical flaw and questioning the transparency and repeatability of our methods; also made us respond in an assertive manner since our scientific integrity was being questioned.*

*That said, we apologize and assure you that in the future we will be more moderated in our response to reviewers. Alas, we do thank you and apologize for this unnecessary exchange. Your comments did improve our manuscript and we are extremely grateful. Again, apologies for any misunderstandings.*

I do not believe that the authors’ response to my comment about lacking definitions of terms such as WUI was adequate. The authors suggest the reader go read another paper to understand what WUI is and why it’s important. If the authors want to only speak to a forestry audience, why do they not publish this piece in a forestry journal?......Instead, the authors could add a few sentences stating what the WUI is, the population that lives there, and that they are relatively more vulnerable to impacts from natural disasters such as wildfires.

*Interestingly WUI is definitely “forestry jargon” which you requested we avoid. However, we have now included several sentences in the Introduction to define this area and why it is important: “The WUI is defined as ““the area where houses exist at more than 1 housing unit per 40 acres and (1) wildland vegetation covers more than 50% of the land area or (2) wildland vegetation covers less than 50% of the land area, but a large area (over 1,235 acres) covered with more than 75% wildland vegetation is within 1.5 miles” (Stewart et al., 2007, p.204). This area is important in wildfire management for two reasons: 1) due to the human population there will be more human caused ignitions and thus wildfires, and 2) wildfires occurring in this area pose a greater risk to structures and lives than one in the forest or more rural areas, are harder to fight, and cannot be left to burn (Radeloff et al. 2018).” Lines 82-89.* *We have added a new reference accordingly.*

My other major concern is that the manuscript is very long, and the writing is not concise. The text could be significantly cut (perhaps by 30-40%) with attention to conciseness. I defer to the editor on this issue.

*Please see our responses to the editor’s requests regarding these same issues. I believe you issues have now been addressed.*
